# Association between Ala379Val polymorphism of lipoprotein-associated phospholipase A2 and migraine without aura in Iranian population

**Published:** 2016-04-03

**Authors:** Faraidoon Haghdoost, Mahsa Gharzi, Farough Faez, Elinaz Hosseinzadeh, Mohamadhasan Tajaddini, Laleh Raﬁei, Fatemeh Asgari, Mahboobeh Banihashemi, Samaneh Sadat Masjedi, Alireza Zandifar, Shaghayegh Haghjooy-Javanmard

**Affiliations:** 1 Medical Student Research Center AND Physiology Research Center, Isfahan University of Medical Sciences, Isfahan, Iran; 2 Medical Student Research Center, Isfahan University of Medical Sciences, Isfahan, Iran; 3 Pharmacy Student Research Center, Isfahan University of Medical Sciences, Isfahan, Iran; 4 Physiology Research Center, Isfahan University of Medical Sciences, Isfahan, Iran

**Keywords:** Headache, Lp-PLA2, Migraine, Gene Polymorphism, Questionnaires‎

## Abstract

**Background:** Migraine is a common neurovascular disorder with multifactorial and polygenic inheritance. The aim of this study was to investigate the association of a migraine without aura and Ala379Val polymorphism of lipoprotein-associated phospholipase A2 (Lp-PLA2) gene in the Iranian population.

**Methods:** In this study, 103 migraine patients and 100 healthy controls were enrolled. DNA samples were extracted and the Ala379Val polymorphism of Lp-PLA2 gene was investigated. To assess severity of a headache, patients filled out the headache impact test (HIT-6) and migraine severity (MIGSEV) questionnaires.

**Results:** Allele V had significantly lower frequency in the case group than control subjects [P = 0.001, odds ratio (OR) = 0.25, confidence interval (CI): 0.15-0.40]. The frequency of migraine patients that were a carrier of V allele (V/V and A/V) was statistically significant lower than the control group (P = 0.003, OR = 2.39, CI: 1.35-4.23). There was no significant difference of alleles frequency between three grades of MIGSEV (P = 0.316). Furthermore, total HIT-6 score was not significantly different between different genotypes (P = 0.466).

**Conclusion:** Our results showed that Ala379Val gene polymorphism of LP-PLA2 is associated with lower risk of migraine but not with severity of headaches in an Iranian population.

## Introduction

A migraine, a major cause of chronic headaches, is a common neurovascular disorder with multifactorial and polygenic inheritance. It affects about 12% of the adult population which is more prevalent in females.^1^^,^^2^ Migraine is recurrent unilateral pulsatile headache and can be accompanied by nausea, vomiting, phonophobia, and photophobia.^3^ Furthermore, some patients have transient neurologic attacks known as “aura.”^4^ The pathogenesis of the migraine is still unclear, but evidence suggests role of inflammation and vascular dysfunction in pain generation.^5^

Lipoprotein-associated phospholipase A2 (Lp-PLA2), also known as platelet-activating factor (PAF) acetylhydrolase, is a calcium-independent serine lipase involved in so many physiological actions and is produced by inflammatory cells such as macrophages, monocytes, and T-lymphocytes.^6^^,^^7^ It is known that mass and activity of Lp-PLA2 associated with race (white is more than black) and sex (in male is more than female).^8^ Lp-PLA2 is an enzyme that circulates in blood and classified as a new inflammatory marker and atherosclerosis risk factor because of the production of oxidized free fatty acids and lysophosphatidylcholines during oxidation of low-density lipoprotein.^6^^,^^9^ The proatherogenic effect of Lp-PLA2 may be through its role in the generation of lysophosphatidylcholine and oxidized fatty acids and also hydrolysis of the proinflammatory mediator PAF.^10^ Lp-PLA2 activity and mass were correlated with increased ultrasound determined carotid intima-media thickness. Moreover, there is an association between Lp-PLA2 and cardiovascular diseases.^7^^,^^8^

The Lp-PLA2 gene is located in the 6 q21.2-p12 chromosome and different polymorphisms have been described for this gene. Studies have shown that some polymorphisms exist in one race that is not shown in others. Val279Phe and Gln281Arg are two polymorphisms of this gene that have only seen in Japanese populations; however, Arg92His, Ile198Thr and Ala379Val, other polymorphisms of this gene have been described more in European populations.^11^^,^^12^ The different polymorphisms of Lp-PLA2 have association with some diseases. For example, G994T has an association with polycystic ovary syndrome and also has increased susceptibility to oxidative stress and inflammation.^13^

Studies have shown that V279F variant in the Lp-PLA2 gene is associated with the increase of myocardial/cerebral infection and also shown an association between this variant and an increased of coronary artery disease (CAD) and infarction. On the other hand, some other polymorphism of this gene like V379 allele of the Ala379Val polymorphism reduced the risk of CAD.^14^ By reducing the Lp-PLA2 protein production, the risk of coronary heart disease (CHD) may decrease. Studies have shown that in individuals homozygous for Ala379Val this enzyme production had reduced and it may lead to less risk for CHD.^15^ As migraine is a vascular disorder and Lp-PLA2 has some vascular effects, the aim of this study was to investigate the association of migraine without aura and Ala379Val polymorphism in Iranian population.

## Materials and Methods

We conducted a case-control study enrolling patients that were recently diagnosed as without aura migraines based on the International Headache Society (IHS) criteria^16^ and also controls that were matched for age, education, sex, and socio-economic status with the cases. The patients were selected from three outpatient neurology clinics between November 2011 and June 2012 in Isfahan, Iran. The socio-demographic and headache characteristics of all the subjects including age, sex, level of education (with and without academic degrees), residency (urban/rural), frequency of headaches, the effect of menstruation on the migraine, and positive family history were asked. Patients who had at least a 3-month history of headaches before the diagnoses and had not have received any medications for their migraine were consecutively enrolled. Controls were selected from healthy people who accompanied the patients that were referred to the neurologic clinics (including patients with migraine and other neurologic disorders) who did not have any history for migraines and also any family history of migraine in the first degree relatives. An informed consent was taken from participants before entering the study. 

***Headache impact test (HIT-6) questionnaire***

To assess the severity of headaches, each patient filled HIT-6 questionnaire. HIT-6 questionnaire consists of six questions that show six dimensions: pain severity, role functioning, social functioning, vitality, emotional distress, and cognitive functioning. Each question has 5 available answers: never, rarely, sometimes, very often, and always. Choices have been scored between 6 and 13. The total score is between 36 and 78.^17^

***Migraine severity (MIGSEV) questionnaire***

Each migraine patient completed an MIGSEV questionnaire as a valid scale for assessing headache severity. The MIGSEV scale, developed by EL Hasnaoui in 2003,^18^ is a simple severity scale with four items including intensity of pain, disability in daily activity, tolerability and nausea that categorize patients in three groups of intensity: mild, moderate, and severe. This instrument is highly reliable, reproducible and sensitive, and we have used its Persian translation in our previous study as a valid scale.^18^^,^^19^

***DNA extraction and genotyping***

A value of 2 ml of venous blood was collected from each participant. Genomic DNA samples were extracted from peripheral whole blood using the AccuPrep Genomic DNA Extraction kit (Bioneer Inc., Korea) according to the manufacturer’s protocol.

The single-nucleotide polymorphisms (SNPs) rs1051931 (A379V) were identified by the National Center for Biotechnology Information (NCBI) data bank and primers were designed by Beacon Designer 8.00 to flank the coding regions (PREMIER Biosoft International, USA and synthesized by TIB MOLBIOL, Germany). 

The forward primer was 5’-TTCTCTTTTAGGGGTTCAGT-3’ and reverse primer was 5’-CATCTGGTTTAGGTCATGAAAA-3’. 

Genotyping was done by high-resolution melt (HRM) assay using a Rotor-Gene 6000 instrument (Corbett Life Science, Australia). Polymerase chain reactions (PCRs) were carried out in duplicate in 20 µl of final volume using the type-it HRM kit (Qiagen), HRM PCR buffer, HotStarTaq Plus DNA Polymerase, nucleotides and EvaGreen dye, and 30 ng DNA. 

The PCR program consisted of an initial denaturation-activation step at 95 °C for 5 minutes, followed by a 40-cycle program (denaturation at 95 °C for 15 seconds, annealing conditions 55 °C for 5 seconds, 72 °C for 15 seconds; a HRM step from 70 to 95 °C rising at 0.1 °C per second). Curves for each duplicate were checked on the shape and peak height to meet reproducibility. Normalized and temperature-shifted melting curves from HRM, suggestive of SNPs, were distinguished and the samples were subjected to direct sequencing.

We analyzed our data with SPSS software (version 18, SPSS Inc., Chicago, IL, USA). An independent t-test was used for quantitative variables between two groups. The relation between polymorphism (homozygous and heterozygous) and different categorized variables, (age, sex, case-control) was established using chi-square test and calculation of odds ratio (OR) [confidence interval (CI) = 95%]. The significant level was considered as P < 0.050.

The study was approved by the Ethical Committee of Isfahan University of Medical Sciences.

## Results

In this study, 103 subjects with migraine and 100 healthy subjects were enrolled, and DNA samples were analyzed for LP-PLA2 Ala379Val gene polymorphisms. In the case and control group, 82.6 and 78.0% were female, respectively, and distribution was not statistically significant. Total mean age of the subjects was 34.42 ± 0.73 (34.08 ± 1.01 and 34.77 ± 1.06 in the case and control groups, respectively, P = 0.639). There were no significant differences in the mean ages, education level (40.0 vs. 36.0% without academic degrees) and residency (51.5 vs. 57.6% urban) between case and control groups, respectively. The migraine characteristics of the case group are shown in table 1.

**Table 1:** The migraine characteristics of the patients

**Characteristics**	**Value**
Frequency of headache per month (mean ± SE)	8.32 ± 0.81
Age onset of migraine (years) (mean ± SE)	26.28 ± 0.94
Family history of migraine [n (%)]	
Yes	64 (77.1)
No	19 (22.9)
Menstrual effect [n (%)]	
Yes	42 (51.9)
No	39 (48.1)
Total HIT-6 score (mean ± SE)	53.51 ± 1.93
MIGSEV grade [n (%)]	
Grade I	9 (12.9)
Grade II	27 (38.6)
Grade III	34 (48.6)

HIT-6: Headache impact test; SE: Standard error; MIGSEV: Migraine severity

The analysis of allele frequency in the case group shows no significant difference in the distribution of A and V alleles in the two genders (P = 0.282), but in control group, V allele frequency in male subjects was significantly more than females (P = 0.008). Another analysis in the case group showed that the distribution of alleles A and V was not associated with family history of migraine and also menstruation effect (P = 0.209 and P = 0.516, respectively). The patients were classified into three groups according to the MIGSEV grade. There was no significant difference of alleles frequency between three grades of MIGSEV (P = 0.316).

The total frequency of allele A and V in the study population were 287 and 119, respectively. Allele V had lower frequency in the case group than control subjects, and the difference was statistically significant (P < 0.001, OR = 0.25) (Table 2). The frequency of A/A, A/V and V/V genotypes in all subjects were 117, 53 and 33, respectively.

The frequency of migraine patients that were carrier of V allele was statistically significant lower than control group (P = 0.003, OR = 2.39, 95% CI: 1.35-4.23). 

Table 3 shows comparison of mean age of onset, frequency of headache per month and total HIT-6 score between A/A and A/V genotypes of LP-PLA2 Ala379Val gene polymorphism in patients. Our results revealed that there are no significant differences between the mean of mentioned factors in the two genotypes (P > 0.050).

**Table 2:** Distribution of allele and genotype of LP-PLA2 Ala379Val gene polymorphism in the case and control groups

**Variable**	**Case [n (%)]**	**Control [n (%)]**	**P**	**OR (95% CI)**
Allele				
A	173 (84)	114 (57)	< 0.001	0.25 (0.15-0.40)
V	33 (16)	86 (43)		
Genotype				
A/A	70 (68)	47 (47)	0.003	2.39 (1.35-4.23)
A/V	33 (32)	20 (20)	0.051	1.88 (0.99-3.58)
V/V	0 (0)	33 (33)	0.000	2.53 (2.10-3.05)

LP-PLA2: Lipoprotein-associated phospholipase A2; OR: Odds ratio; CI: Confidence interval

## Discussion

As migraine is a vascular disorder and Lp-PLA2 has some vascular effects, the aim of this study was to investigate the association of migraine without aura and Ala379Val polymorphism in Iranian population. Our results showed that frequency of V allele (mutant allele) was lower in case group than control group. We classified our subjects into three genotypes of A/A, A/V, and V/V. The frequency of migraine in the subjects who are carrier of V allele was statistically significant lower than non-V allele carriers (OR = 2.39, CI: 1.35-4.23), so the current study suggests that V allele of Ala379Val polymorphism of LP-PLA2 will be related to decreased risk for migraine. We also investigated the association of Ala379Val polymorphism with severity and frequency of headaches in migraine patients. Our results revealed no association between the polymorphism and mentioned factors. To the best of our knowledge, there is no study conducted to assess the association of Ala379Val polymorphism of LP-PLA2 and migraine. But according to results of a study conducted on the genetic of migraine although they could not find a relation between Lp-PLA2 gene and migraine, but they have reported that a predisposing haplotype spanning 10 Mb on the chromosome 6p12.2-p21.1 that contains the Lp-PLA2 gene, was inherited with all migraine patients in the pedigree that they have studied.^20^

There are some other studies designed to assess association of other disorders and Ala379Val polymorphism of LP-PLA2. Abuzeid et al. in their study have reported that homozygosity for the V379 allele was associated with lower risk of myocardial infarction (MI), but the risk of MI in the AA and AV subjects was very similar.^15^ Similar to this report, our results showed that there was no significant difference between AA and AV groups in the frequency of migraine. However, VV genotype was associated with lower risk of migraine. Results of another study conducted by Oei et al. revealed that activity of Lp-PLA2 is associated with risk of CHD and also ischemic stroke.^21^ This report confirms the Abuzeid et al.^15^ conclusion as we know that V379 allele is associated with lower activity of Lp-PLA2 protein.^21^

In a study by Kardara et al.^22^ that was designed to investigate the association of Ala379Val polymorphism with hypertension and thrombotic markers, results showed that A/V genotype is associated with lower risk of essential hypertension. But both A/A and V/V genotypes were at higher risk of developing essential hypertension. Their results also showed that in hypertensive patients, A/V genotype carriers have elevated level of fibrinogen. They have reported that in hypertensive patients, A/V genotype is associated with increased inﬂammatory and thrombotic burden.

## Conclusion

Our results showed that Ala379Val gene polymorphism of LP-PLA2 is associated with lower risk of migraine without aura in Iranian population. However, it has no effect on the severity of migraine and also frequency of headaches in migraine patients. We had a small sample size and it reduced the power of our study especially in analysis of association of the polymorphism with frequency of headache and severity of migraine. Therefore, this is a preliminary conclusion. Further studies with larger sample sizes are needed.

**Table 3:** Comparison of mean age of onset, frequency of headache per month and total headache impact test (HIT-6) score between A/A and A/V genotypes in patients

**Variable**	**Genotype**		**P**
	**A/V**	**A/A**	
	**Mean ± SE**	**Mean ± SE**	
Age of onset	28.56 ± 1.88	25.38 ± 1.08	0.131
Frequency of headache per month	7.86 ± 1.70	8.49 ± 0.93	0.739
Total HIT-6 score	51.24 ± 3.77	54.40 ± 2.26	0.466

HIT-6: Headache impact test; SE: Standard error

## Notes:

**How to cite this article:** Haghdoost F, Gharzi M, Faez F, Hosseinzadeh E, Tajaddini M, Raﬁei L, et al. Association between Ala379Val polymorphism of lipoprotein-associated phospholipase A2 and migraine without aura in Iranian population. Iran J Neurol 2016; 15(2): 80-4.
